# *TP53*-specific mutations serve as a potential biomarker for homologous recombination deficiency in breast cancer: a clinical next-generation sequencing study

**DOI:** 10.1093/pcmedi/pbae009

**Published:** 2024-04-09

**Authors:** Yongsheng Huang, Shuwei Ren, Linxiaoxiao Ding, Yuanling Jiang, Jiahuan Luo, Jinghua Huang, Xinke Yin, Jianli Zhao, Sha Fu, Jianwei Liao

**Affiliations:** Cellular & Molecular Diagnostics Center, Sun Yat-sen Memorial Hospital, Sun Yat-sen University, Guangzhou 510120, China; Guangdong Provincial Key Laboratory of Malignant Tumor Epigenetics and Gene Regulation, Sun Yat-sen Memorial Hospital, Sun Yat-sen University, Guangzhou 510120, China; Department of Clinical Laboratory, The Sixth Affiliated Hospital, Sun Yat-sen University, Guangzhou 510655, China; Breast Tumor Center, Sun Yat-sen Memorial Hospital, Sun Yat-sen University, Guangzhou 510120, China; Guangzhou Regenerative Medicine and Health, Guangdong Laboratory, Sun Yat-sen Memorial Hospital, Sun Yat-sen University, Guangzhou 510120, China; Cellular & Molecular Diagnostics Center, Sun Yat-sen Memorial Hospital, Sun Yat-sen University, Guangzhou 510120, China; Cellular & Molecular Diagnostics Center, Sun Yat-sen Memorial Hospital, Sun Yat-sen University, Guangzhou 510120, China; Cellular & Molecular Diagnostics Center, Sun Yat-sen Memorial Hospital, Sun Yat-sen University, Guangzhou 510120, China; Cellular & Molecular Diagnostics Center, Sun Yat-sen Memorial Hospital, Sun Yat-sen University, Guangzhou 510120, China; Breast Tumor Center, Sun Yat-sen Memorial Hospital, Sun Yat-sen University, Guangzhou 510120, China; Guangzhou Regenerative Medicine and Health, Guangdong Laboratory, Sun Yat-sen Memorial Hospital, Sun Yat-sen University, Guangzhou 510120, China; Cellular & Molecular Diagnostics Center, Sun Yat-sen Memorial Hospital, Sun Yat-sen University, Guangzhou 510120, China; Cellular & Molecular Diagnostics Center, Sun Yat-sen Memorial Hospital, Sun Yat-sen University, Guangzhou 510120, China

**Keywords:** next-generation sequencing, homologous recombination deficiency, *TP53*, breast cancer

## Abstract

**Background:**

*TP53* mutations and homologous recombination deficiency (HRD) occur frequently in breast cancer. However, the characteristics of *TP53* pathogenic mutations in breast cancer patients with/without HRD are not clear.

**Methods:**

Clinical next-generation sequencing (NGS) of both tumor and paired blood DNA from 119 breast cancer patients (BRCA-119 cohort) was performed with a 520-gene panel. Mutations, tumor mutation burden (TMB), and genomic HRD scores were assessed from NGS data. NGS data from 47 breast cancer patients in the HRD test cohort were analyzed for further verification.

**Results:**

All *TP53* pathogenic mutations in patients had somatic origin, which was associated with the protein expression of estrogen receptor and progestogen receptor. Compared to patients without *TP53* pathologic mutations, patients with *TP53* pathologic mutations had higher levels of HRD scores and different genomic alterations. The frequency of *TP53* pathologic mutation was higher in the HRD-high group (HRD score ≥ 42) relative to that in the HRD-low group (HRD score < 42). *TP53* has different mutational characteristics between the HRD-low and HRD-high groups. *TP53*-specific mutation subgroups had diverse genomic features and TMB. Notably, *TP53* pathogenic mutations predicted the HRD status of breast cancer patients with an area under the curve (AUC) of 0.61. TP53-specific mutations, namely HRD-low mutation, HRD-high mutation, and HRD common mutation, predicted the HRD status of breast cancer patients with AUC values of 0.32, 0.72, and 0.58, respectively. Interestingly, *TP53* HRD-high mutation and HRD common mutation combinations showed the highest AUC values (0.80) in predicting HRD status.

**Conclusions:**

*TP53*-specific mutation combinations predict the HRD status of patients, indicating that *TP53* pathogenic mutations could serve as a potential biomarker for poly-ADP-ribose polymerase (PARP) inhibitors in breast cancer patients .

## Introduction

Breast and ovarian cancers frequently present homologous recombination deficiency (HRD), making them susceptible to poly-ADP-ribose polymerase inhibitors (PARPi), a novel cancer treatment designed for such malignancies based on the concept of synthetic death. [1]. Numerous studies have identified *BRCA1* and *BRCA2* (*BRCA1/2*) as important components of homologous recombination (HR), a specific DNA repair mechanism, in which mutations in these genes frequently lead to HRD in breast cancers [2, 3]. Although there are also many works suggesting that mutations in the HR genes contribute to HRD, *TP53*-specific characterization and its effect on HRD in breast cancer is not clear.

Recently, *TP53* mutations have frequently been observed in breast cancer patients, who exhibit traits linked to proliferative, aggressive behavior, and poor clinical outcomes [4]. For example, Cosgrove *et al*. identified that *TP53* mutations were enriched in breast cancer with brain metastasis [5]. The aggressive breast cancer phenotype is usually associated with a genetic alteration in *TP53* [6]. However, Wang *et al*. found that *TP53* mutations correlate with a high rate of complete pathological remission in response to to anthracycline/cyclophosphamide neoadjuvant chemotherapy in breast cancer patients [7]. The reason for these contradictory results may be due to differences in breast cancer molecular subtypes and *TP53* mutation types. Therefore, in the real world, a more in-depth characterization of *TP53* mutations in breast cancer is particularly important.

The essential functions of *TP53* in controlling important cellular processes have been well documented, including cell cycle arrest, apoptosis, DNA repair, and genomic stability [8–10]. BRCAness refers to HRD due to defects in the HR-associated non-*BRCA1/2* genes and is an important marker for the treatment of breast cancer based on synthetic lethal PARPi [11]. A subset of breast cancer patients with *TP53* mutation shares numerous distinguishing characteristics of “BRCAness” [12]. Due to faulty DNA repair mechanisms, these tumors have a high incidence of *TP53* deletions and insertions, which increases their susceptibility to DNA breaks [12]. Indeed, more than one-third of DNA damage repair genes (including *TP53* and *BRCA1/2*) have mutations accompanied by heterozygous deletions in human cancers including breast cancer [13]. However, the *TP53*-specific characterization and clinical significance of *TP53* pathogenic mutations in breast cancer patients with/without HRD remain unknown.

Here, we analyzed the characteristics of *TP53* pathogenic mutations in breast cancer patients with/without HRD using the clinical next-generation sequencing (NGS) data. Obviously, *TP53* pathogenic mutations are associated with HRD scores and different genomic alterations. Notably, a specific mutation signature of *TP53* is presented in patients with HRD. Furthermore, *TP53*-specific mutation combinations (HRD-high mutations and HRD common mutations) predict the HRD status of patients, which indicates that *TP53* pathogenic mutations may serve as potential biomarkers for PARPi in breast cancer patients.

## Materials and methods

### Patients

With patient-informed agreement, 119 breast cancer patients (BRCA-119 cohort) and 47 breast cancer patients (HRD test cohort) from the Sun Yat-sen Memorial Hospital were enrolled in this study. Between May 2022 and April 2023, 119 patients at Sun Yat-sen Memorial Hospital had breast cancer tissue and matched normal blood samples collected. Age, gender, immunohistochemistry for estrogen receptor (ER), progestogen receptor (PR), Her2, Ki67, and genetic tests were all collected retrospectively to get the medical record. Pathological diagnosis of the patients was completed by at least two certified pathologists. All NGS experiments and data analysis were performed in the Cellular & Molecular Diagnostics Center of the hospital. The Institutional Review Board of Sun Yat-sen Memorial Hospital, Sun Yat-Sen University, allowed this study (NO. SYSKY-2023–458-01).

### DNA extraction and NGS

Genomic DNA was isolated from FFPE tissues and matched normal blood following the manufacturer's instructions using the DNeasy Blood and DNA FFPE tissue kit (Qiagen, Germany). The OncoScreen Plus panel used in the BRCA-119 cohort comprises 520 genes and includes ∼9000 single-nucleotide polymorphisms (SNPs) that are evenly distributed across the human genome. Table S1 (see online supplementary material) displays a list of the 520 genes associated with cancer. NGS was performed with 20 homologous recombination repair (HRR)-related genes (supplementary Fig. S1, see online supplementary material) and numerous SNPs across the human genome from patients from the HRD test cohort. The NGS experiments were conducted as previously described [14]. To create an NGS library, at least 50 ng of high-quality DNA was required. The tissue DNA underwent end repair, phosphorylation, and adapter ligation after being sheared. The 200–400 bp DNA fragments from the sheared tissue were isolated using an Agencourt AMPure XP kit. Then, hybrid capture, magnetic-bead purification, and PCR amplification were carried out. After the NGS library was created, the indexed samples were sequenced using Illumina NextSeq 550 sequencing apparatus (Illumina, USA) utilizing paired-end reads and a 1000 average sequencing depth.

### NGS data analysis

NGS data analysis was completed as previously described [14]. The readings were rapidly mapped by BWA Picard to the hg19 reference human genome [15]. Gene variants were found using VarScan and the Genome Analysis Tool Kit [16,17]. Locates with a depth of <100 were filtered out using the VarScan fpfilter pipeline. Base calling in plasma and tissue samples needed at least eight supporting reads for single nucleotide mutations, but only two and five supporting reads for insertion–deletion variants. Compared with white blood cells, somatic variants of tumor tissue were discovered. The variants were annotated by both ANNOVAR and SnpEff [18, 19]. FACTERA was used for the DNA translocation investigation [20]. Finally, two knowledgeable technicians confirmed all identified mutations using the Integrative Genomics Viewer system [21].

### Variation identification and classification

Sequence data were analyzed using specialized computational methods to reliably detect somatic and germline variations while separating sequencing artifacts from true positive mutations. Variants with a population frequency > 0.1% were classified as SNPs and excluded from further study using information from the 1000 Genomes, ExAC, and dbSNP databases. A total of 71 genes associated with tumor inheritance were included in germline variations analysis [22]. The clinical significance of each variation was evaluated using the 5-tier classification system of: pathogenic/oncogenic, likely pathogenic/oncogenic, benign, likely benign, and variants of uncertain significance following the standards of the Clinical Genome Resource, the American College of Medical Genetics and Genomics, the Association for Molecular Pathology, and the internal pipeline [23–26]. For those variants without accessible conclusions from expert panels, ClinVar's consensus classifications were employed. Benign and likely benign variations were not included in the subsequent analysis. For the classification of *TP53* variants, we defined pathogenic/oncogenic and likely pathogenic/oncogenic as pathogenic mutations.

### Tumor mutation burden estimation

The total number of discovered non-synonymous mutations divided by the entire coding region size of the panel used to generate the panel's output was used to determine the tumor mutation burden (TMB) per patient [14]. Using the following equation, we calculated TMB as a ratio: TMB = the total number of nonsynonymous mutations (except for SNPs and hot mutations)/1.003 Mb [27].

### HRD score calculation

The ∼9000 SNPs that are evenly distributed across the human genome are utilized for HRD score estimation. An in-house script, named Burning Rock Instability Detection of the Genome (BRIDGE) was developed for this purpose, and was previously described by Feng *et al*. [27]. The loss of heterozygosity (LOH) score was defined as the number of LOH patches >15 Mb but smaller than the whole chromosome [28]. The number of chromosomal breaks between adjacent areas that are ≥10 Mb apart and have a distance of ≤3 Mb are known as large-scale state transitions (LST) [29]. Telomeric allelic imbalance (TAI) refers to the quantity of uneven parental allele contributions that extend to the telomere ends of chromosomes [30]. NGS data were generated using these three scores [31]. Genomic scar scores were estimated by the sum of LOH, LST, and TAI [31, 32].

### Statistical analysis

R software and GraphPad Prism were also used for statistical analysis. To investigate the mutation frequency in distinct groups, Fisher's exact test was used. The differences in HRD, LOH, LST, TAI, and TMB between the two groups were compared using the Mann–Whitney U test, while the differences between several groups were compared using analysis of variance. A significance level of *P* < 0.05 was used for statistical tests.

## Results

### *TP53* pathologic mutations and clinicopathologic factors in breast cancer patients

Among 119 patients, most patients (∼80%) were young. *TP53* pathogenic variants were present in 68 patients, and all of these variants were of somatic cell origin. According to the Chinese Society of Clinical Oncology 2023 guidelines, we divided these patients into four subtypes: luminal A, luminal B, HER2+, and triple-negative breast cancer (TNBC). The percentage of these four subtypes is 3.36% of luminal A, 55.46% of luminal B, 21.85% of HER2+, and 15.97% of TNBC (supplementary Fig. S2A, see online supplementary material). Notably, *TP53* pathologic mutation occurs in ∼60% of all patients, about 50% of luminal B, about 70% of HER2+, and about 90% of TNBC (supplementary Fig. S2B). However, there was no difference in the percentage of *TP53* pathologic mutation types among these subtypes (supplementary Fig. S2C,D). Notably, LOH, LST, TAI, and their sum HRD score were inordinately different among these four subtypes (supplementary Fig. S3, see online supplementary material). Moreover, patients with TNBC had the highest LOH, LST, TAI, and HRD scores among the four subtypes (supplementary Fig. S3).

Next, we divided the patients into the *TP53* pathogenic mutant group and non-*TP53* pathogenic mutant (without *TP53* pathogenic mutations) group. The clinicopathologic factors and *TP53* pathogenic mutations in breast cancer patients are shown in Table 1. Notably, *TP53* pathogenic mutation was associated with the protein expression of ER and PR, as well as subtypes (Table 1). This suggested that *TP53* pathogenic mutations have a significant role in the molecular characterization of breast cancer.

**Table 1.** *TP53* pathologic mutations and clinical factors in breast cancer patients.

Clinical factor	Numbers	Non-*TP53* pathologic mutation	*TP53* pathologic mutation	*P* value
Total	119	51	68	
Age, years				0.7416
<60	95	40	55	
≥60	24	11	13	
ER expression				*P* < 0.001
<1% (+)	26	5	21	
1–10% (+)	15	2	13	
>10% (+)	74	41	33	
Not available	4	3	1	
PR expression				*P* < 0.001
<1% (+)	48	10	38	
1–10% (+)	17	10	7	
>10% (+)	50	28	22	
Not available	4	3	1	
Her2 expression				0.0816
Negative	89	41	48	
Positive	26	7	19	
Not available	4	3	1	
Ki67 expression				0.2204
<14% (+)	10	6	4	
≥14% (+)	105	42	63	
Not available	4	3	1	
Subtypes				*P* < 0.001
Luminal A	4	4	0	
Luminal B	66	35	31	
HER2+	26	7	19	
TNBC	19	2	17	
Not available	4	3	1	
T stage				0.5339
T0–T2	40	12	28	
T3–T4	10	3	7	
Tx	3	0	3	
Not available	66	36	30	
N stage				0.8981
N0	16	5	11	
N1–2	22	6	16	
N3–4	9	3	6	
Nx	6	1	5	
Not available	66	36	30	
M stage				0.6583
M0	38	11	27	
M1	13	4	9	
Mx	2	0	2	
Not available	66	36	30	

### *TP53* pathologic mutations are associated with high genomic scar scores

**Figure 1.**
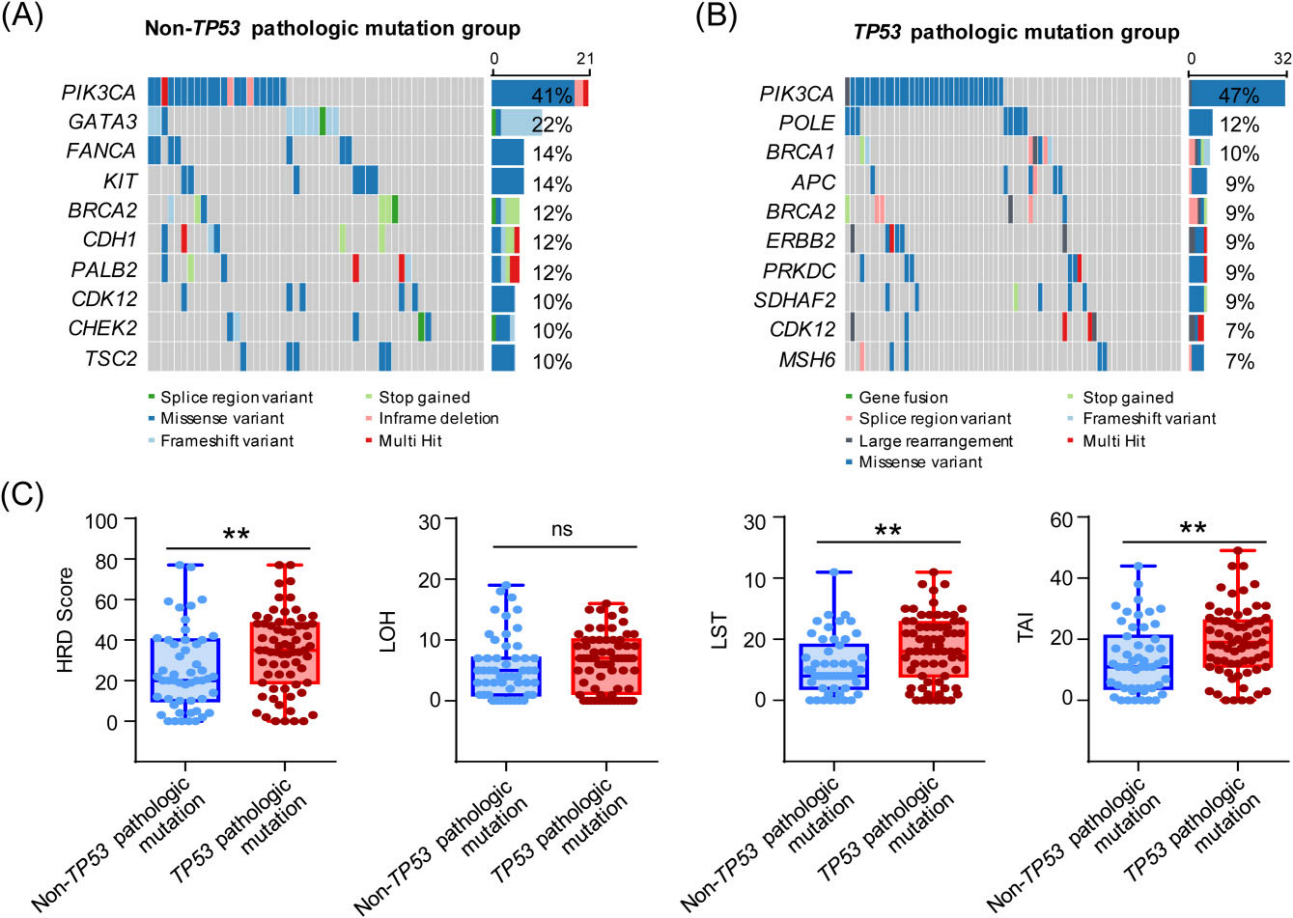
*TP53* pathologic mutations are associated with genomic scar scores. (**A**) Mutation frequency and characterization of the top 10 genes in the non-*TP53* pathologic mutation group. (**B**) Mutation frequency and characterization of the top 10 genes in the TP53 pathologic mutation group. (**C**) Genomic scar scores in the non-*TP53* pathologic mutation and TP53 pathologic mutation groups. **P < 0.01; ns, not significant.

Since *TP53* pathologic mutations are associated with the protein expression of ER and PR, as well as subtypes (Table 1), we questioned whether *TP53* pathologic mutations cause genomic alterations. To address this, we analyzed the genomic features of the *TP53* pathologic mutant and non-*TP53* pathologic mutant groups, respectively. In the non-*TP53* pathologic mutant group, the top 10 mutated genes are *PIK3CA* (41%), *GATA3* (22%), *FANCA* (14%), *KIT* (14%), *BRCA2* (12%), *CDH1* (12%), *PALB2* (12%), *CDK12* (10%), *CHEK2* (10%), and *TSC2* (10%) (Fig. 1A). However, in the *TP53* pathologic mutant group, the top 10 mutated genes are *PIK3CA* (47%), *POLE* (12%), *BRCA1* (10%), *APC* (9%), *BRCA2* (9%), *ERBB2* (9%), *PRKDC* (9%), *SDHAF2* (9%), *CDK12* (7%), and *MSH6* (7%) (Fig. 1B). Among the top 10 mutated genes, only two genes (*PIK3CA* and *BRCA2*) overlapped in the *TP53* pathologic mutant and non-*TP53* pathologic mutant groups. Furthermore, *CDH1* and *GATA2* had higher frequencies in the *TP53* pathologic mutant group, whereas *SDHF2* had a higher frequency in the non-*TP53* pathologic mutant group (Table S2), suggesting that the genomic alterations were not the same in the two groups.

As *TP53* pathologic mutations cause genomic alterations, we investigated whether such mutations are associated with HRD in breast cancer. We therefore calculated genomic scar scores (including LOH, LST, TAI, and their sum) for both groups of patients from the NGS data. Notably, the *TP53* pathologic mutant group had higher HRD scores relative to the non-*TP53* pathologic mutant group (Fig. 1C). Although *TP53* pathologic mutations were not associated with LOH, compared to the non-*TP53* pathologic mutant group, patients with *TP53* pathologic mutations had higher levels of LST and TAI (Fig. 1C). To verify this, we analyzed our internal HRD test cohort and found that the *TP53* pathologic mutant group also had higher HRD scores relative to the non-*TP53* pathologic mutant group (supplementary Fig. S4A, see online supplementary material). For further verification, the publicly available whole exome sequencing data and HRD scores of the Cancer Genome Atlas (TCGA) breast cancer patients were obtained from the study by Thorsson *et al*. [33]. Consistently, breast cancer patients with *TP53* pathologic mutations had significantly higher HRD scores than patients without *TP53* pathologic mutations (supplementary Fig. S4B). These results suggest that TP53 pathologic mutations are associated with HRD in breast cancer.

### Characterization of *TP53* pathologic mutations in the low and high HRD score groups

**Figure 2.**
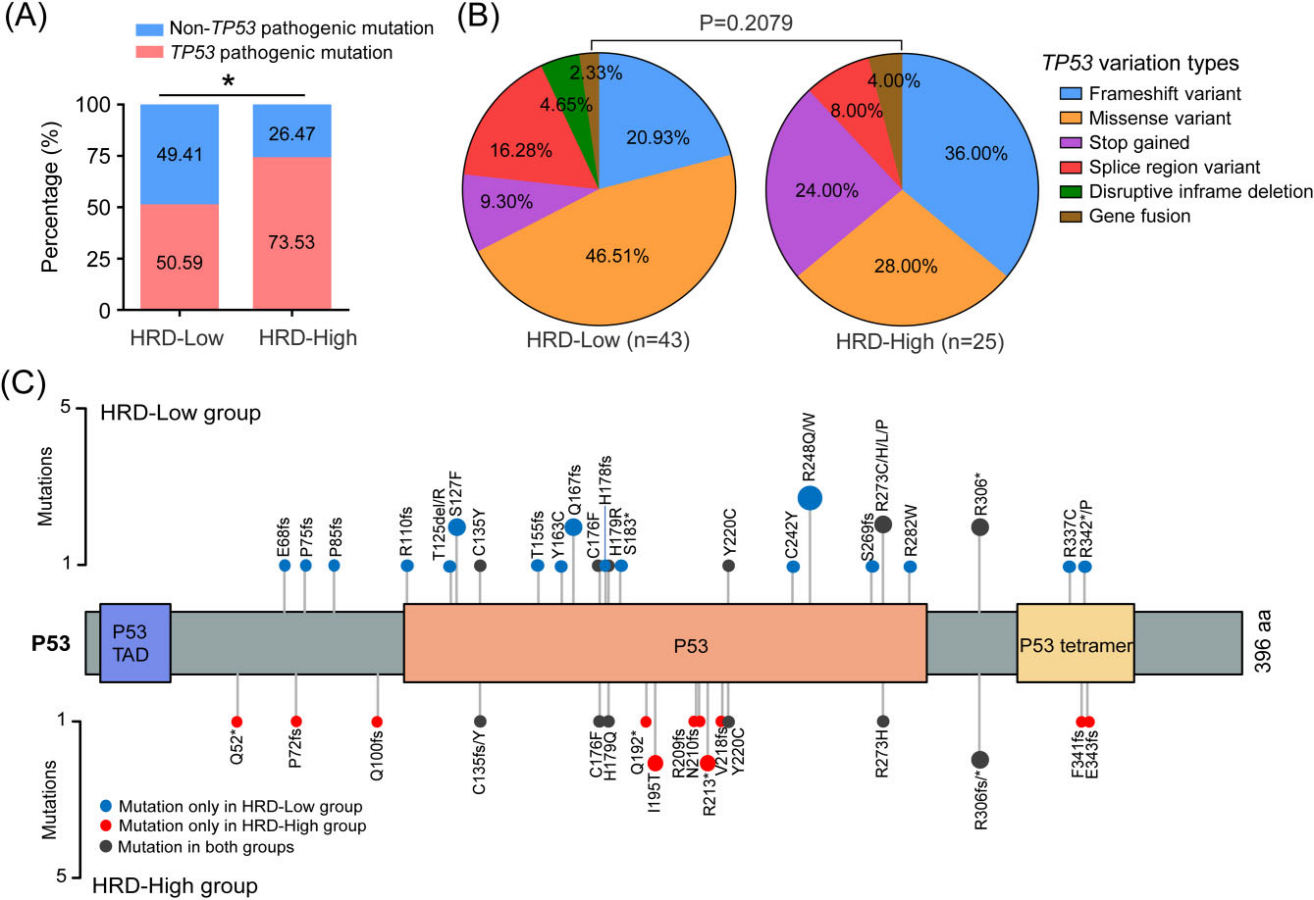
*TP53* mutation characterization in the low and high HRD score groups. (**A**) Proportion of *TP53* pathologic mutations in the low and high HRD score groups. (**B**) *TP53* pathologic mutation type in the low and high HRD score groups. (**C**) Lollipop chart of *TP53* coding-region mutations in the low and high HRD score groups.*P < 0.05.

To explore the mutational characterization of *TP53* in patients with breast cancer, we categorized patients into HRD-low (HRD score < 42) and HRD-high (HRD score ≥ 42) groups based on HRD scores, which is the level for HRD in ovarian cancer that has previously been reported [32]. Obviously, the frequency of *TP53* pathologic mutation was higher in the HRD-high group relative to that in HRD-low group (Fig. 2A). However, the distribution of *TP53* pathologic mutation types (including frameshift variant, missense variant, nonsense variation, splice region variation, inframe deletion, and gene fusion) did not differ between the two groups (Fig. 2B). We next analyzed the HRD scores among different *TP53* pathogenic mutation type groups. Although LOH was higher in the stop gained group than in the splice variant and other type groups, HRD score showed no difference among *TP53* pathogenic mutation type groups (supplementary Fig. S5, see online supplementary material). Moreover, similar *TP53* pathologic mutation frequency, mutation type distribution, and HRD scores among different groups were verified in the HRD test cohort (supplementary Fig. S6, see online supplementary material).

Detailed information on *TP53* pathologic mutations (including splice region variation, inframe deletion, and gene fusion) is shown in Table 2. It is noteworthy that two mutations of *TP53* (ATP1B2-TP53, c.375 + 1dup) appeared only in the HRD-high group, while one mutation (c.376–1G > A) was present in both groups (Table 2). In the protein-coding region, 17 mutation sites of *TP53* appeared only in the HRD-low group, while 11 mutation sites occurred only in the HRD-high group. Furthermore, there are six mutation sites present in both groups (Fig. 2C). These results indicated that *TP53* has different mutational characteristics between the HRD-low and HRD-high groups.

**Table 2.** Detailed information on *TP53* splice region variation, inframe deletion, and gene fusion.

Description	Mutation type	Classification	HRD score	Allele fraction
*ATP1B2-TP53*	Gene fusion	Likely pathologic	44	9.50%
c.375 + 1dup	Splice region variant	Pathologic	68	60.00%
c.376–1G > A	Splice region variant	Pathologic	61	22.59%
c.376–1G > A	Splice region variant	Pathologic	19	49.72%
c.559 + 1G > A	Splice region variant	Pathologic	8	16.76%
c.559 + 1G > T	Splice region variant	Pathologic	0	3.82%
c.783–1G > T	Splice region variant	Likely pathologic	5	9.39%
c.97–2A > C	Splice region variant	Likely pathologic	19	37.05%
SHBG-TP53	Gene fusion	Likely pathologic	40	47.99%
p.E336_R337del	Inframe deletion	Likely pathologic	12	10.58%
p.T230_I232del	Inframe deletion	Likely pathologic	4	12.38%

### Different *TP53*-specific mutation subgroups with diverse genomic features

**Figure 3.**
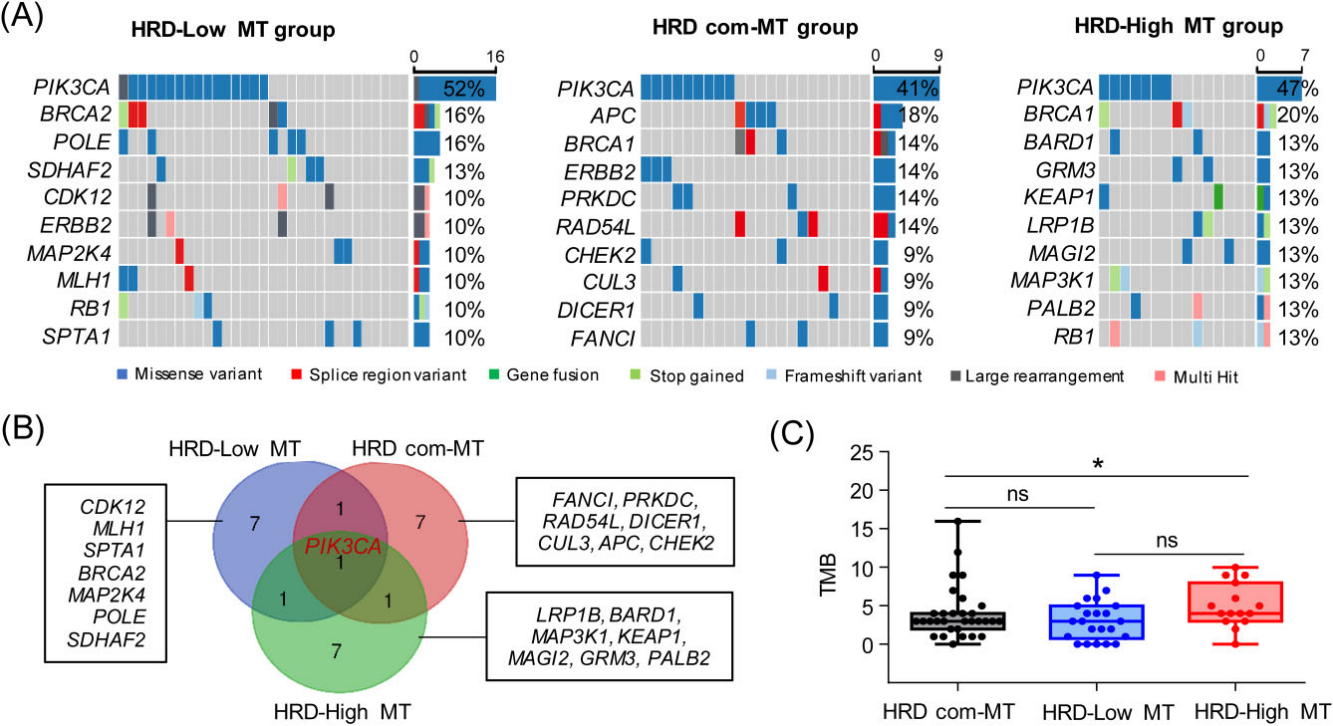
Genomic characterization based on grouping of different *TP53*-specific mutations. (**A**) Mutation frequency and characterization of the top 10 genes in the different *TP53*-specific mutation groups. (**B**) Venn diagram of the top 10 genes in the different *TP53*-specific mutation groups. (**C**) Distribution of TMB in the indifferent *TP53*-specific mutation groups. *P < 0.05; ns, not significant.

To investigate the impact of the *TP53* pathologic mutation on the genome, we categorized the *TP53* pathologic mutations based on HRD. *TP53* pathologic mutations that appeared only in the HRD-low group were denoted as HRD-low specific mutations, the mutations that occurred only in the HRD-high group were defined as HRD-high specific mutations, while mutations present in both groups were defined as HRD common mutations. According to these *TP53* pathologic mutations, we categorized *TP53* pathologic mutated patients into HRD-low mutation (HRD-low MT), HRD-high mutation (HRD-high MT), and HRD common mutation (HRD com-MT) groups. We then analyzed the genomic profiles of these three groups and found that there are significant differences in the genomic alterations among the three groups (Fig. 3A). Among the top 10 mutated genes, only *PIK3CA* overlapped in the groups, while seven genes appeared only in their subgroups, respectively (Fig. 3B). To further confirm these results, the publicly available whole exome sequencing data of breast cancer patients were obtained from TCGA. According to the *TP53* pathologic mutation site in this study, we categorized breast cancer patients into HRD-high MT, HRD-low MT, and HRD com-MT groups. Notably, there are also significant differences in the genomic alterations among the three groups (supplementary Fig. S7A, see online supplementary material). Moreover, among the top 10 mutated genes, only *TTN* and *PIK3CA* were overlapping in the three groups (supplementary Fig. S7B, see online supplementary material). Using TMB as a response indicator of mutation in the genome, we next analyzed the TMB situation for the three groups. Notably, there was a significant difference in TMB among the three groups (Fig. 3C). This suggests that *TP53*-specific mutation might be associated with the genomic alteration.

### *TP53*-specific mutation combinations predict HRD status

**Figure 4.**
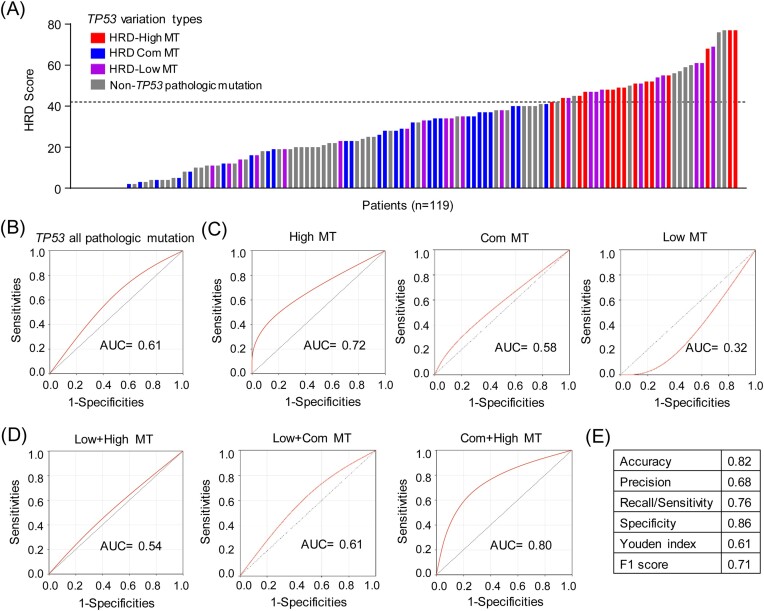
*TP53*-specific mutation combinations predict HRD status. (**A**) Distribution of *TP53*-specific mutations in different subgroups with different HRD scores. (**B**) ROC curves for *TP53* pathologic mutations in predicting HRD status. (**C**) ROC curves for different *TP53*-specific mutations in predicting HRD status. (**D**) ROC curves for different combinations of *TP53*-specific mutations in predicting HRD status. (**E**) A variety of indicators for evaluating the performance of *TP53*-specific (Com + High) mutation prediction. HRD-Low MT, *TP53* mutations specific in the HRD-Low group; HRD-High MT, *TP53* mutations specific in the HRD-High group; HRD com-MT, *TP53* mutations both in the HRD-low and HRD-High groups; *TP53*-specific (Com + High) mutation, *TP53* with HRD com-MT or HRD-High MT; AUC, area under the curve; ROC, receiver operating characteristic.

To further pursue the associations between *TP53* pathologic mutations and HRD, we demonstrated the distribution of HRD score and *TP53*-specific mutation in breast cancer patients (Fig. 4A). A total of 34 breast cancer patients had HRD scores >42 (HRD-high score), which is the level for HRD in ovarian cancer that has previously been documented [32]. Among these 34 breast cancer patients, 25 patients harbored a pathogenic mutation in *TP53* (Fig. 4A).

Since *TP53* pathologic mutations are associated with high HRD scores, we then pondered whether *TP53* pathologic mutations could be used to predict breast cancer HRD status. Interestingly, *TP53* pathogenic mutations predicted HRD status of breast cancer patients with an AUC of 0.61 (Fig. 4B). Notably, specific mutations, namely HRD-low MT, HRD-high MT, and HRD com-MT, predicted HRD status of breast cancer patients with AUC values of 0.32, 0.72, and 0.58, respectively (Fig. 4C). To further improve the sensitivity and specificity, we performed different combinations of *TP53*-specific mutations. Notably, HRD-high MT and HRD com-MT combinations showed the highest AUC values (0.80) in predicting HRD status (Fig. 4D). To comprehensively evaluate the prediction performance, other indicators including accuracy, precision, recall/sensitivity, specificity, Youden index, and F1 score were calculated. The confusion matrix of true and predicted values is shown in supplementary Fig. S8, see online supplementary material. Notably, this model has reasonable predictive performance, with an accuracy of 0.82, precision of 0.68, recall/sensitivity of 0.76, specificity of 0.86, Youden index of 0.61, and F1 score of 0.71 (Fig. 4E). These results indicated that *TP53*-specific mutation combinations predict the HRD status of breast cancer patients.

## Discussion

Recently, although PARPi and platinum-based chemotherapy significantly improved the survival of breast cancer patients with HRD, whether *TP53*-specific mutated patients would obtain benefits from PARPi treatment is unknown. Here, we collected real-world clinical NGS data and evaluated the association between *TP53* pathologic mutation and HRD status in breast cancer patients. As an important focus, *TP53* pathogenic mutations are associated with HRD scores and different genomic alterations. This first comprehensive analysis identified that the combination of *TP53*-specific mutations (HRD-high MT and HRD com-MT) predicts the HRD status of patients and may serve as a potential biomarker for PARPi in breast cancer patients.

Previous studies of *TP53* in cancer mainly focused on cell cycle arrest, apoptosis, DNA repair, and genomic stability [8–10]. Recently, a few studies have reported that *TP53* mutation is associated with proliferative, aggressive behavior, and poor clinical outcomes in breast cancer [4]. Here, we found that *TP53* pathogenic mutations were associated with the protein expression of ER and PR, as well as subtype (Table 1), suggesting that *TP53* pathogenic mutations have a significant role in the molecular characterization of breast cancer. Indeed, *TP53* pathogenic mutations tend to occur in breast cancers with an aggressive phenotype characterized by poor differentiation, increased invasiveness, and high potential metastasis [4]. For example, *TP53* mutations were enriched in breast cancer with brain metastasis [5]. This fully reflects the important role of *TP53* in breast cancer, especially in molecular phenotypes.

Originally, the concept of BRCAness was used to describe the HRD signatures that occur in BRCA1/2-deficient cancer cells, which are characterized by hypersensitivity to PARPi [34]. Subsequently, several studies have found that mutations in other genes besides *BRCA1/2* can also result in BRCAness, including HR-related genes, DNA damage signaling genes, and Fanconi-anemia-related genes [35]. Many studies now view BRCAness as a synonym of HRD [11, 35]. Notably, recent research revealed that several non-HR genes might additionally trigger HRD in patients with breast cancer [36, 37]. For instance, HRD is synthetically lethal in breast cancer because *ALC1* loss promotes chromosomal instability brought on by DNA gaps that are left unrepaired on duplication spears [36]. *GATA3* is required for HR to repair DNA double-strand breaks in breast cancer [37]. Here, we found that *TP53* pathogenic mutations were associated with HRD in breast cancer patients, which was further verified in our internal HRD test cohort and TCGA cohort. Moreover, *TP53* pathogenic mutations may mainly affect LST and TAI. Indeed, LOH is mainly due to the germline pathogenic variants of *BRCA1/2* in breast cancer [32, 38]. Of course, how *TP53* affects LST and TAI and the molecular mechanisms underlying these processes require further experimental studies.

The most prevalent somatic mutations in *TP53* associated with cancer are point mutations, followed by minor insertions, deletions, and rearrangements [39, 40]. Missense mutations that cover the protein DNA-binding domain are concentrated mostly in exons 5–8, which predominate in the mutation spectrum of all malignancies, including breast cancer [39, 40]. The frameshift mutations appear evenly across the gene and are dispersed across the coding region [39, 40]. Similar to previous studies, in terms of mutation types, the main somatic mutation types of *TP53* in breast cancer were also point mutations, followed by frameshift mutation and rearrangements. Notably, the distribution of *TP53* pathogenic mutation types did not differ between the HRD-low and HRD-high groups, suggesting that the different role of *TP53* in HRD may be due to various mutation positions. Indeed, in the protein-coding region, 17 mutation sites of *TP53* appeared only in the HRD-low group, while 11 mutation sites occurred only in the HRD-high group. Based on this, we first screened for HRD-specific *TP53* mutation sites that are well-predictive of HRD scores.

According to the infinite site model of molecular evolution, a mutation may occur at any given location in the genome only once [41]. However, biallelic mutations in cancer genomes identify local factors that influence mutation [42]. For instance, biallelic mutations in *FANCM* exhibit chromosomal fragility and increase the risk of breast cancer and chemotherapy toxicity [43]. *TP53* biallelic mutations result in the reclassification of sectional acute myeloid leukemia/myelodysplastic syndromes cases from monoallelic to multi-hit [44]. Moreover, *TP53* biallelic mutations are uniquely related to a poorer prognosis in myelodysplastic syndromes [44]. Here, we did not find patients with *TP53* biallelic mutations in breast cancer patients, which is probably due to the small sample size. Although the frequency of *TP53* biallelic mutation is low in breast cancer patients, this may be a prognostic risk factor for breast cancer, which needs clinical validation via large samples in further study.

In clinical practice, the identification of BRCAness/HRD in patients is important but remains complex. The most direct method is to detect HR genes by sequencing, such as *BRCA1, BRCA2, PALB2*, and *RAD51*. The FoundationOne CDx sequenced 324 cancer-related genes, including 16 HR genes [45]. This approach was restricted to harmful homozygous mutations and excluded those parallel pathways in HRD cells, such as 53BP1 inactivation and the restoration of DNA repair. At present, mutation signatures based on whole exome sequencing are limited in clinical application because of numerous objective factors. The second approach is to use Myriad myChoice CDx to calculate HRD scores from the levels of LOH, TAI, and LST [46]. The HRD score is a measure of the genomic scar as a result of past genomic instability. This approach requires covering a wide range of probes and considering the boundaries of HRD scores. The third approach is to induce a measurable RAD51 response *in vitro* by exposure to genotoxic substances and to detect RAD51 filaments by microscopy [47]. However, this functional strategy requires the use of replicating cells and fresh tumor tissue and is still at the experimental stage. A fourth strategy is to exploit the BRCAness/HRD transcriptional signatures on the premise that HR gene deletion leads to adaptations of cellular pathways to compensate for HRD [48]. This approach is potentially attractive because tumors are typically treated by RNA sequencing. All of the above-mentioned methods have their specific shortcomings, which limit their use on a wide scale in clinical practice.

In this study, we constructed a convenient strategy for HRD prediction by *TP53*-specific mutation combinations (HRD-high mutations and HRD common mutations). However, the AUC values were unsatisfactory in the HRD test cohort and TCGA patients. This may be due to the different coverage of NGS detection for *TP53*. Although our study has some limitations, the combination of *TP53*-specific mutations to a certain extent predicted the patient's HRD status. This greatly reduces the number of sequenced genes and has an excellent predictive power for HRD. Nevertheless, this is only at the level of the prediction of HRD status. Unfortunately, due to the limitations of current data in this study, the platinum/PAPRi therapy efficiency of these *TP53* pathogenic mutation groups is indistinct. Therefore, whether this model can be used for the clinical efficacy of platinum/PAPR inhibitor treatment in breast cancer patients needs to be verified in future studies.

## Conclusion

In conclusion, our study identified that *TP53* pathogenic mutations were associated with HRD scores. Patients with HRD had *TP53*-specific mutation signatures. Furthermore, a combination of *TP53*-specific mutations (HRD-high mutations and HRD common mutations) predicted the patient's HRD status, which may serve as potential biomarkers for PARPi in breast cancer.

## Ethical declaration

The study was conducted in accordance with the Helsinki Declaration. Patient records were anonymized prior to analysis. This study was approved by the Institutional Review Board of Sun Yat-sen Memorial Hospital (NO. SYSKY-2023–458-01).

## Supplementary Material

pbae009_Supplemental_File
