# Supplementary material for: TP53-specific mutations serve as a potential biomarker for homologous recombination deficiency in breast cancer: a clinical next-generation sequencing study
Source: Precis Clin Med. 2024 Apr 9;7(2):pbae009. doi: 10.1093/pcmedi/pbae009 (PMC11092399; doi:10.1093/pcmedi/pbae009)
Supplement: pbae009_Supplemental_File [file pbae009_supplemental_file.pdf]

TP53-specific mutations serve as a potential biomarker for homologous recombination deficiency in breast cancer: a clinical next-generation sequencing study

Yongsheng Huang *et al.*

Supplementary Material
Figures S1 and S8
Tables S1 – S2

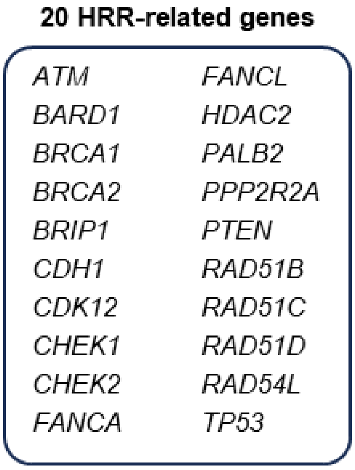

Figure S1. The 20 HRR-related genes were detected in our internal HRD test cohort. HRR, homologous recombination repair.

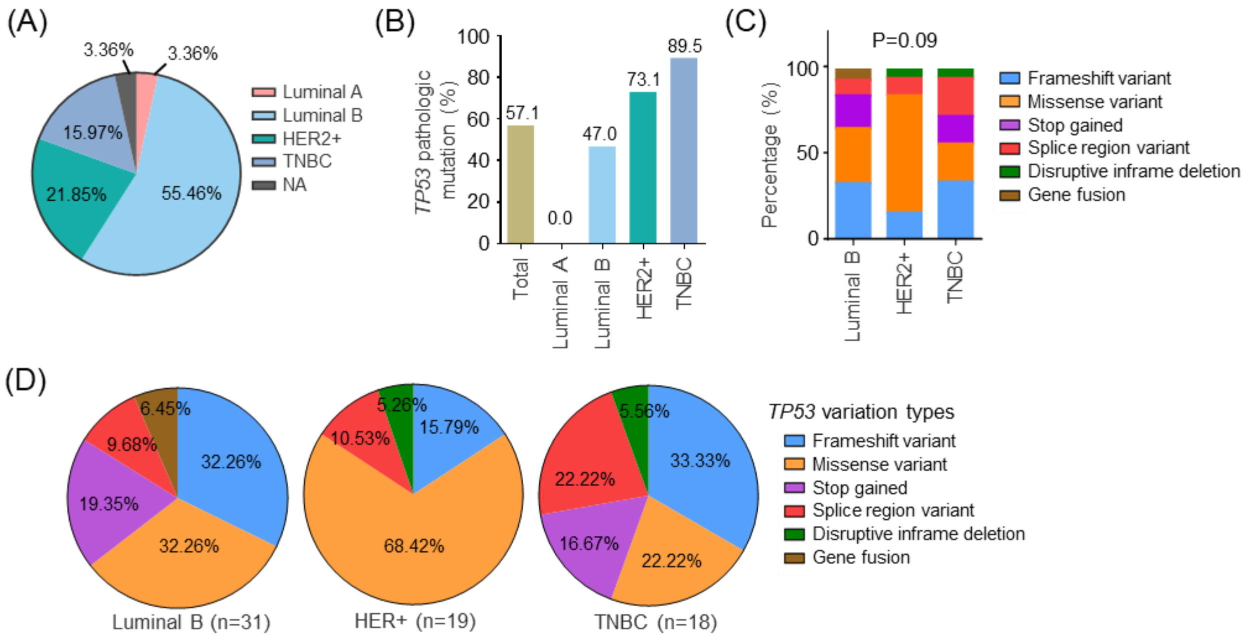

Figure S2. *TP53* pathologic mutation characteristics in different breast cancer subgroups.

(A) The percentage of patients with different subgroups of breast cancer in the BRCA-119 cohort. (B) The *TP53* pathologic mutation frequency in different breast cancer subgroups. (C-D) *TP53* pathologic mutation types in different breast cancer subgroups. HER2+, patients with positive HER2 expression; TNBC, Triple-negative breast cancer.

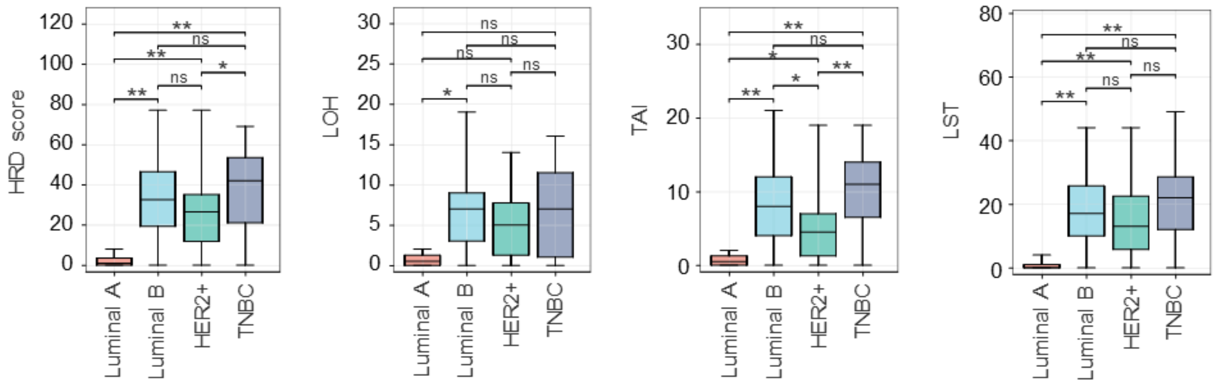

Figure S3. Homologous recombination deficiency in different breast cancer subgroups.

Genomic scar scores between different breast cancer subgroups in the BRCA-119 cohort. HRD, Homologous recombination deficiency; LOH, Loss of heterozygosity; LST, Large-scale state transition; TAI, Telomeric allelic imbalance. *, P<0.05; **, P<0.01; ns, not significant; HER2+, patients with positive HER2 expression; TNBC, Triple-negative breast cancer.

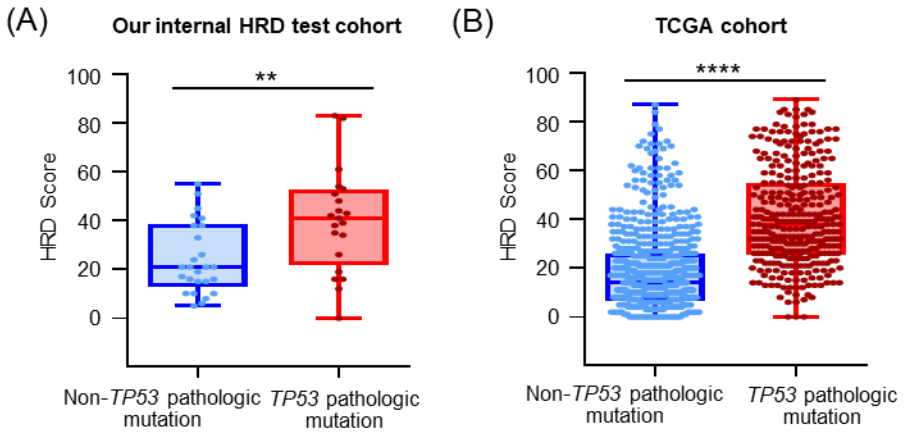

Figure S4. Homologous recombination defects in different groups in the validation cohort. (A) Boxplots exhibiting the geographic distribution of homologous recombination defects score between Non-*TP53* pathologic mutation and *TP53* pathologic mutation groups in our internal HRD test cohort. (B) Homologous recombination defects in different groups in the TCGA BRCA cohort. HRR, homologous recombination repair; TCGA, The Cancer Genome Atlas; BRCA, Breast cancer; HRD, Homologous recombination deficiency. **, P<0.01; ****, P<0.0001.

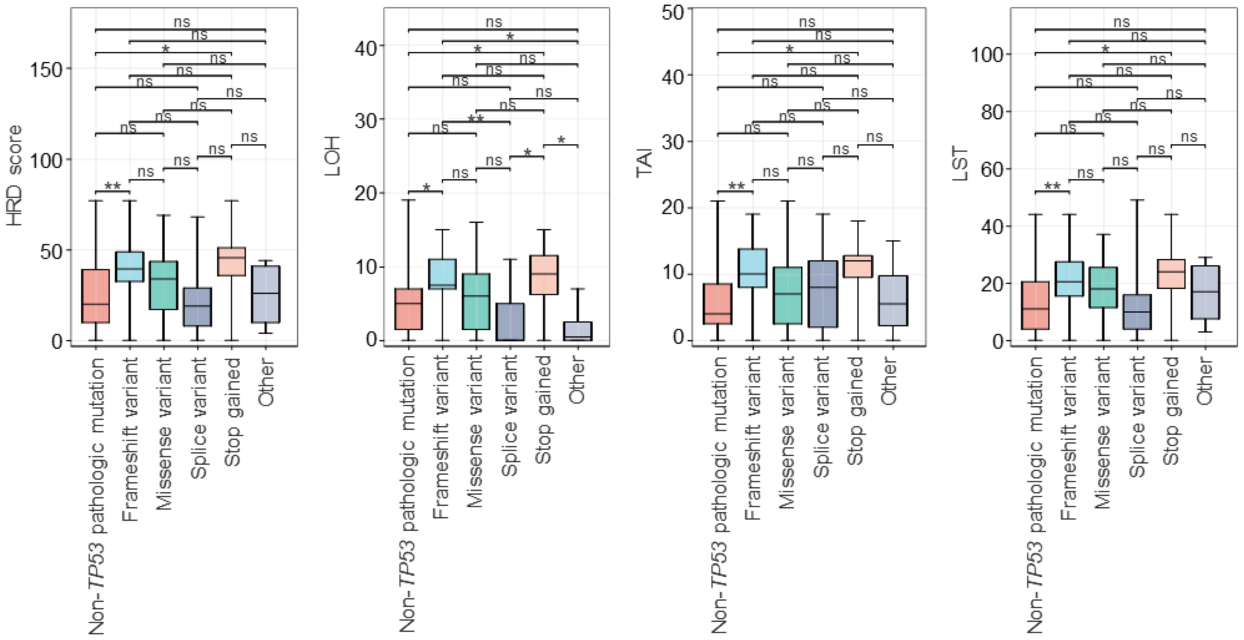

Figure S5. Homologous recombination deficiency in different *TP53* status groups.

Genomic scar scores in the Non-*TP53* pathologic mutation and different *TP53* pathologic mutation type groups. HRD, Homologous recombination deficiency; LOH, Loss of heterozygosity; LST, Large-scale state transition; TAI, Telomeric allelic imbalance. *, P<0.05; **, P<0.01; ns, not significant.

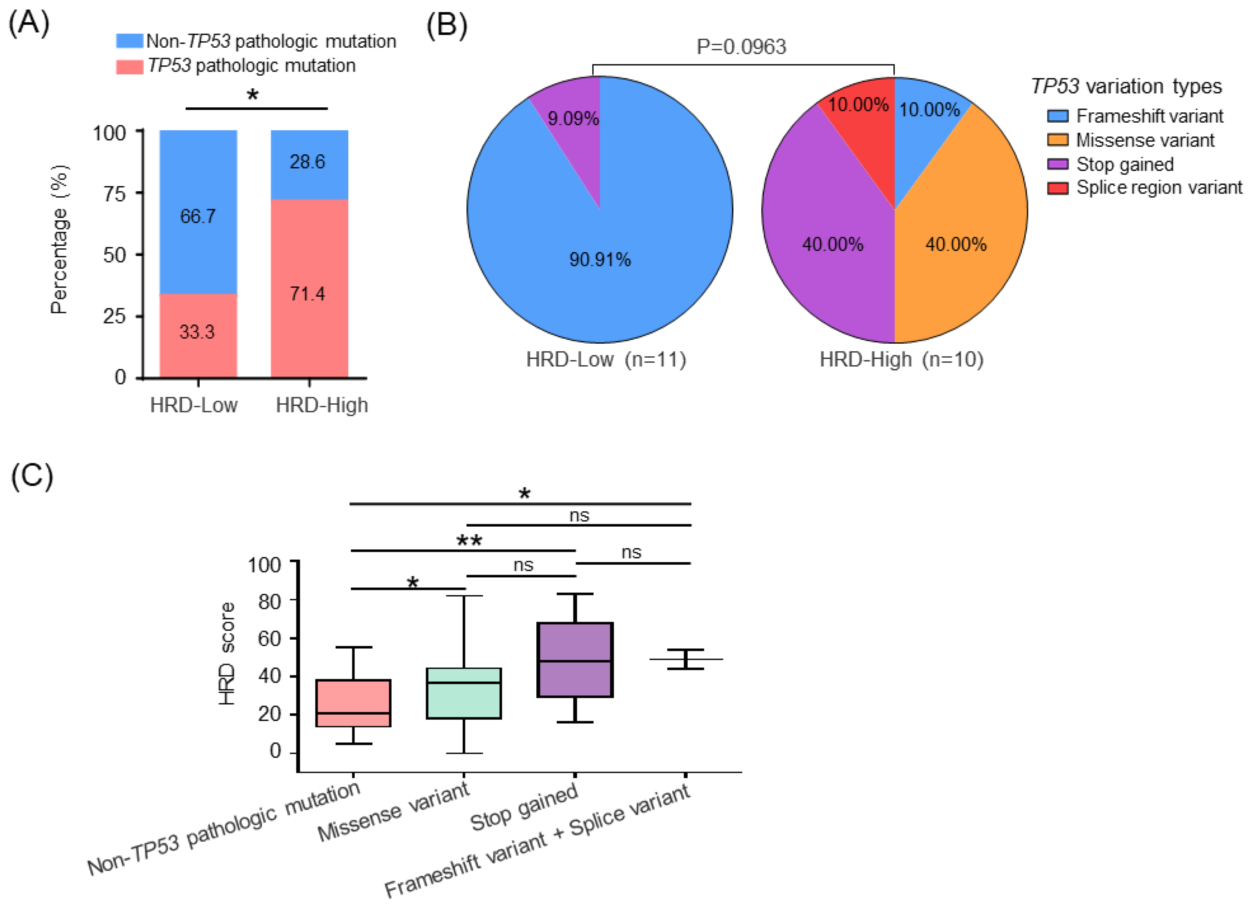

Figure S6. *TP53* pathologic mutation characterization in our internal HRD test cohort.

(A) BRCA patients with *TP53* mutation and HRD score data from our internal HRD test cohort were analyzed. *TP53* pathologic mutation frequency in HRD-Low and HRD-High groups. (B) HRD score in the Non-*TP53* pathologic mutation and different *TP53* pathologic mutation type groups. (C) The lollipop chart of *TP53* coding-region mutations in the HRD-Low and HRD-High groups. BRCA, Breast cancer; HRD, Homologous recombination deficiency. *, P<0.05; **, P<0.01; ns, not significant.

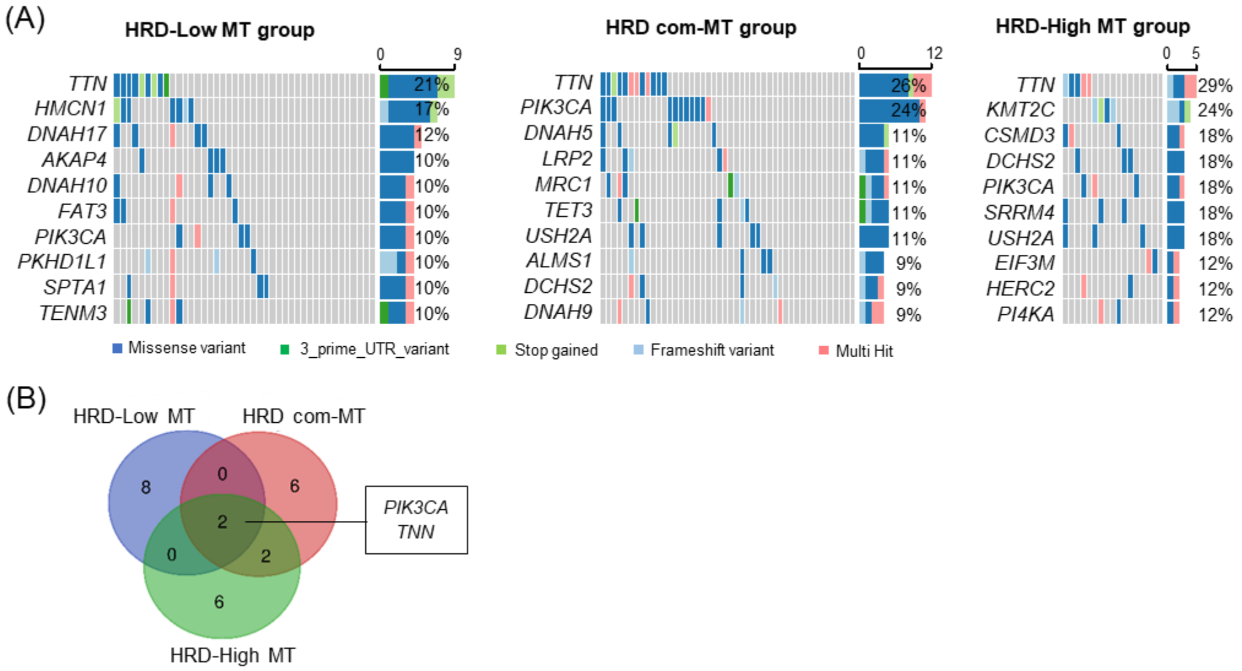

Figure S7. Genomic characterization between different *TP53*-specific mutations in the TCGA BRCA cohort.

(A) The mutation frequency and characterization of the top 10 genes in the different *TP53*-specific mutation groups. (B) Venn diagram of top 10 genes in the different *TP53*-specific mutation groups. HRD-Low MT, *TP53* pathologic mutations specific in the HRD-low group; HRD-High MT, *TP53* pathologic mutations specific in the HRD-High group; HRD com-MT, *TP53* pathologic mutations both in the HRD-low and HRD-High groups; TCGA, The Cancer Genome Atlas; BRCA, Breast cancer.

Predicted value \ True value	HRD-High	HRD-Low
HRD-High	25	12
HRD-Low	9	73

Figure S8. The confusion matrix of true and predicted values.

Table S1. The 520 gene list.

ABL1	NM_005157.5	*KLF4*	NM_001314052.1
AKT1	NM_001014432.1	*KRAS*	NM_033360.3
AMER1	NM_152424.3	*LTK*	NM_002344.5
ARID1A	NM_006015.4	*MAP2K1*	NM_002755.3
ASXL2	NM_018263.4	*MAPK1*	NM_002745.4
AURKB	NM_001284526.1	*MDM2*	NM_002392.5
BAP1	NM_004656.3	*MERTK*	NM_006343.2
BCL2L1	NM_001317919.1	*MKNK1*	NM_003684.5
BCORL1	NM_021946.4	*MSH2*	NM_000251.2
BRCA1	NM_007294.3	*MTAP*	NM_002451.3
BRIP1	NM_032043.2	*MYCN*	NM_001293228.1
CARD11	NM_032415.5	*NCOA3*	NM_181659.2
CCND2	NM_001759.3	*NF2*	NM_000268.3
CD79A	NM_001783.3	*NOTCH1*	NM_017617.4
CDK12	NM_016507.3	*NRAS*	NM_002524.4
CDKN1B	NM_004064.4	*NT5C2*	NM_001134373.2
CEBPA	NM_004364.4	*NUP93*	NM_014669.4
CHEK1	NM_001274.5	*PAK5*	NM_020341.3
CRLF2	NM_022148.3	*PAX5*	NM_016734.2
CTCF	NM_006565.3	*PDGFRA*	NM_006206.4
CUL4A	NM_001008895.2	*PIK3C2B*	NM_002646.3
DAXX	NM_001141970.1	*PIK3CD*	NM_005026.3
DIS3	NM_014953.4	*PIM1*	NM_001243186.1
DOT1L	NM_032482.2	*PNRC1*	NM_006813.2
EIF4E	NM_001130679.1	*PPP2R1A*	NM_014225.5
EPHA3	NM_005233.5	*PRKAR1A*	NM_002734.4
ERBB2	NM_004448.3	*PTEN*	NM_000314.6
ERCC3	NM_000122.1	*PTPRT*	NM_133170.3
ESR1	NM_000125.3	*RAD50*	NM_005732.3
EZH2	NM_004456.4	*RAD52*	NM_001297419.1
FANCE	NM_021922.2	*RB1*	NM_000321.2
FANCM	NM_020937.3	*RHEB*	NM_005614.3
FGF12	NM_021032.4	*ROS1*	NM_002944.2
FGF4	NM_002007.2	*RSPO2*	NM_178565.4
FGFR3	NM_000142.4	*SDHAF2*	NM_017841.2
FLT3	NM_004119.2	*SF3B1*	NM_012433.3
FOXP1	NM_001244810.1	*SLC34A2*	NM_006424.2
GATA1	NM_002049.3	*SMAD4*	NM_005359.5
GEN1	NM_001130009.2	*SNCAIP*	NM_001308100.1
GNAQ	NM_002072.4	*SOX9*	NM_000346.3
GRM3	NM_000840.2	*SRSF2*	NM_003016.4
H3F3C	NM_001013699.2	*STAT5B*	NM_012448.3

HIST1H2BD	NM_021063.3	*TAF1*	NM_001286074.1
HIST1H3G	NM_003534.2	*TENT5C*	NM_017709.3
HIST3H3	NM_003493.2	*TGFBR1*	NM_001306210.1
HOXB13	NM_006361.5	*TNFAIP3*	NM_001270507.1
ID3	NM_002167.4	*TP63*	NM_003722.4
IGF1R	NM_000875.4	*TSC1*	NM_000368.4
IL7R	NM_002185.3	*UGT1A1*	NM_000463.2
INSR	NM_000208.3	*WRN*	NM_000553.4
JAK1	NM_001320923.1	*XRCC3*	NM_001100118.1
KDM5A	NM_001042603.2	*ZNF217*	NM_006526.2
KEL	NM_000420.2	*ACVR1*	NM_001105.4
KMT2C	NM_170606.2	*ALK*	NM_004304.4
LMO1	NM_002315.2	*ARAF*	NM_001256196.1
MAGI2	NM_012301.3	*ARID5B*	NM_032199.2
MAP3K1	NM_005921.1	*ATRX*	NM_000489.4
MCL1	NM_021960.4	*AXL*	NM_021913.4
MEF2B	NM_001145785.1	*BCL10*	NM_003921.4
MIR21	NR_029493.1	*BCL6*	NM_001130845.1
MPL	NM_005373.2	*BMPR1A*	NM_004329.2
MST1	NM_020998.3	*BRD7*	NM_001173984.2
MYC	NM_002467.4	*BTK*	NM_000061.2
NAV3	NM_001024383.1	*CBL*	NM_005188.3
NEGR1	NM_173808.2	*CD274*	NM_014143.3
NKX2-1	NM_001079668.2	*CDH1*	NM_004360.4
NOTCH4	NM_004557.3	*CDK8*	NM_001260.2
NSD2	NM_001042424.2	*CDKN2B*	NM_004936.3
NTRK2	NM_006180.4	*CHD2*	NM_001271.3
PAK1	NM_001128620.1	*CREBBP*	NM_004380.2
PARP2	NM_005484.3	*CSMD1*	NM_033225.5
PDCD1	NM_005018.2	*CTNNB1*	NM_001904.3
PGR	NM_000926.4	*CYP17A1*	NM_000102.3
PIK3CA	NM_006218.3	*DDR2*	NM_001014796.1
PIK3R2	NM_005027.3	*DNMT3A*	NM_022552.4
PMS1	NM_000534.4	*EGFR*	NM_005228.3
PPARG	NM_015869.4	*EPCAM*	NM_002354.2
PRDM1	NM_001198.3	*EPHB1*	NM_004441.4
PRKN	NM_004562.2	*ERCC1*	NM_202001.2
PTPRO	NM_030667.2	*ERG*	NM_001136154.1
RAC1	NM_018890.3	*ETV6*	NM_001987.4
RAD51C	NM_058216.2	*FANCC*	NM_000136.2
RARA	NM_000964.3	*FANCI*	NM_001113378.1
REL	NM_002908.3	*FBXW7*	NM_033632.3
RIT1	NM_001256821.1	*FGF23*	NM_020638.2
RPS6KB2	NM_003952.2	*FGFR1*	NM_023110.2

SDC4	NM_002999.3	*FLCN*	NM_144997.5
SDHD	NM_003002.3	*FOXL2*	NM_023067.3
SH2D1A	NM_002351.4	*FYN*	NM_002037.5
SMAD2	NM_001003652.3	*GATA4*	NM_001308093.1
SMARCD1	NM_003076.4	*GNA11*	NM_002067.4
SOX17	NM_022454.3	*GREM1*	NM_013372.6
SPTA1	NM_003126.2	*H3F3A*	NM_002107.4
STAT4	NM_001243835.1	*HGF*	NM_000601.5
SUFU	NM_016169.3	*HIST1H3D*	NM_003530.4
TCF7L2	NM_001146274.1	*HIST1H3J*	NM_003535.2
TET1	NM_030625.2	*HLA-C*	NM_001243042.1
TMEM127	NM_017849.3	*HSP90AA1*	NM_001017963.2
TOP2A	NM_001067.3	*IFNGR1*	NM_000416.2
TRIM58	NM_015431.3	*IKZF1*	NM_006060.5
TYRO3	NM_006293.3	*INPP4A*	NM_001134224.1
VHL	NM_000551.3	*IRS1*	NM_005544.2
XPO1	NM_003400.3	*JUN*	NM_002228.3
ZBTB16	NM_001018011.1	*KDR*	NM_002253.2
ABL2	NM_007314.3	*KLHL6*	NM_130446.2
AKT2	NM_001626.5	*LATS1*	NM_004690.3
APC	NM_000038.5	*LYN*	NM_002350.3
ARID1B	NM_020732.3	*MAP2K2*	NM_030662.3
ATM	NM_000051.3	*MAPK3*	NM_002746.2
AXIN1	NM_003502.3	*MDM4*	NM_002393.4
BARD1	NM_000465.3	*MET*	NM_000245.3
BCL2L11	NM_001204107.1	*MLH1*	NM_000249.3
BIRC3	NM_001165.4	*MSH3*	NM_002439.4
BRCA2	NM_000059.3	*MTOR*	NM_004958.3
BTG1	NM_001731.2	*MYD88*	NM_002468.4
CASP8	NM_001228.4	*NCOR1*	NM_006311.3
CCND3	NM_001760.4	*NFE2L2*	NM_006164.4
CD79B	NM_000626.3	*NOTCH2*	NM_024408.3
CDK4	NM_000075.3	*NRG1*	NM_001322205.1
CDKN1C	NM_000076.2	*NTHL1*	NM_002528.6
CENPA	NM_001809.3	*NUTM1*	NM_001284292.1
CHEK2	NM_007194.3	*PALB2*	NM_024675.3
CSF1R	NM_001288705.1	*PBRM1*	NM_018313.4
CTLA4	NM_005214.4	*PDGFRB*	NM_002609.3
CXCR4	NM_003467.2	*PIK3C2G*	NM_001288772.1
DCUN1D1	NM_020640.3	*PIK3CG*	NM_001282426.1
DNAJB1	NM_006145.2	*PLCG2*	NM_002661.4
DPYD	NM_000110.3	*POLD1*	NM_001256849.1
EMSY	NM_001300942.1	*PPP2R2A*	NM_002717.3
EPHA5	NM_001281765.2	*PRKCI*	NM_002740.5

ERBB3	NM_001982.3	*PTPN11*	NM_002834.3
ERCC4	NM_005236.2	*QKI*	NM_006775.2
ETV4	NM_001079675.2	*RAD51*	NM_001164269.1
EZR	NM_001111077.1	*RAD54L*	NM_003579.3
FANCF	NM_022725.3	*RBM10*	NM_001204468.1
FAS	NM_000043.5	*RHOA*	NM_001664.3
FGF14	NM_175929.2	*RPA1*	NM_002945.3
FGF6	NM_020996.2	*RUNX1*	NM_001754.4
FGFR4	NM_002011.4	*SDHB*	NM_003000.2
FLT4	NM_182925.4	*SGK1*	NM_001143676.1
FRS2	NM_001042555.2	*SLIT2*	NM_004787.3
GATA2	NM_001145661.1	*SMARCA4*	NM_001128849.1
GID4	NM_024052.4	*SOCS1*	NM_003745.1
GNAS	NM_080425.3	*SPEN*	NM_015001.2
GSK3B	NM_002093.3	*STAG2*	NM_001042749.2
HDAC1	NM_004964.2	*STK11*	NM_000455.4
HIST1H3A	NM_003529.2	*TBX3*	NM_016569.3
HIST1H3H	NM_003536.2	*TERC*	NR_001566.1
HLA-A	NM_001242758.1	*TGFBR2*	NM_001024847.2
HRAS	NM_005343.3	*TNFRSF14*	NM_003820.3
IDH1	NM_005896.3	*TRAF2*	NM_021138.3
IGF2	NM_000612.5	*TSC2*	NM_000548.4
INHA	NM_002191.3	*VEGFA*	NM_001025366.2
IRF2	NM_002199.3	*WT1*	NM_024426.4
JAK2	NM_004972.3	*YAP1*	NM_001282101.1
KDM5C	NM_004187.3	*ZNF703*	NM_025069.2
KIT	NM_000222.2	*ACVR1B*	NM_020328.3
KMT2D	NM_003482.3	*ALOX12B*	NM_001139.2
LRP1B	NM_018557.2	*ARFRP1*	NM_001267547.2
MALT1	NM_006785.3	*ASXL1*	NM_015338.5
MAP3K13	NM_001242314.1	*AURKA*	NM_001323303.1
MDC1	NM_014641.2	*B2M*	NM_004048.2
MEN1	NM_000244.3	*BCL2*	NM_000633.2
MITF	NM_000248.3	*BCOR*	NM_001123383.1
MRE11	NM_005591.3	*BRAF*	NM_004333.4
MST1R	NM_002447.3	*BRINP3*	NM_199051.2
MYCL	NM_001033082.2	*CALR*	NM_004343.3
NBN	NM_002485.4	*CCND1*	NM_053056.2
NF1	NM_000267.3	*CD74*	NM_001025159.2
NKX3-1	NM_006167.3	*CDH18*	NM_001291956.1
NPM1	NM_002520.6	*CDKN1A*	NM_001291549.1
NSD3	NM_023034.1	*CDKN2C*	NM_001262.2
NTRK3	NM_001012338.2	*CHD4*	NM_001273.3
PAK3	NM_001128168.2	*CRKL*	NM_005207.3

PARP3	NM_001003931.3	*CSMD3*	NM_198123.1
PDCD1LG2	NM_025239.3	*CUL3*	NM_001257198.1
PHOX2B	NM_003924.3	*CYP2D6*	NM_000106.5
PIK3CB	NM_006219.2	*DICER1*	NM_177438.2
PIK3R3	NM_001303427.1	*DNMT3B*	NM_006892.3
PMS2	NM_000535.6	*EIF1AX*	NM_001412.3
PPM1D	NM_003620.3	*EPHA2*	NM_004431.4
PREX2	NM_024870.3	*EPHB4*	NM_004444.4
PTCH1	NM_000264.3	*ERCC2*	NM_000400.3
PTPRS	NM_002850.3	*ERRFI1*	NM_018948.3
RAD21	NM_006265.2	*EWSR1*	NM_013986.3
RAD51D	NM_002878.3	*FANCD2*	NM_001018115.2
RASA1	NM_002890.2	*FANCL*	NM_018062.3
RET	NM_020975.4	*FGF10*	NM_004465.1
RNF43	NM_017763.5	*FGF3*	NM_005247.2
RPTOR	NM_020761.2	*FGFR2*	NM_000141.4
SDHA	NM_004168.3	*FLT1*	NM_002019.4
SETD2	NM_014159.6	*FOXO1*	NM_002015.3
SHQ1	NM_018130.2	*GABRA6*	NM_000811.2
SMAD3	NM_005902.3	*GATA6*	NM_005257.5
SMO	NM_005631.4	*GNA13*	NM_006572.5
SOX2	NM_003106.3	*GRIN2A*	NM_000833.4
SRC	NM_198291.2	*H3F3B*	NM_005324.4
STAT5A	NM_001288718.1	*HIST1H1C*	NM_005319.3
SYK	NM_001174167.2	*HIST1H3E*	NM_003532.2
TEK	NM_000459.4	*HIST2H3D*	NM_001123375.2
TET2	NM_001127208.2	*HNF1A*	NM_000545.6
TMPRSS2	NM_001135099.1	*ICOSLG*	NM_001283050.1
TP53	NM_000546.5	*IGF1*	NM_001111285.2
TRPC5	NM_012471.2	*IL10*	NM_000572.2
U2AF1	NM_001025203.1	*INPP4B*	NM_001101669.1
WISP3	NM_198239.1	*IRS2*	NM_003749.2
XRCC2	NM_005431.1	*KAT6A*	NM_006766.4
ZBTB2	NM_020861.2	*KEAP1*	NM_012289.3
ABRAXAS1	NM_139076.2	*KMT2A*	NM_001197104.1
AKT3	NM_005465.4	*LATS2*	NM_014572.2
AR	NM_000044.3	*MAF*	NM_005360.4
ARID2	NM_152641.2	*MAP2K4*	NM_001281435.1
ATR	NM_001184.3	*MAX*	NM_002382.4
AXIN2	NM_004655.3	*MED12*	NM_005120.2
BBC3	NM_001127240.2	*MGA*	NM_001164273.1
BCL2L2	NM_001199839.1	*MLH3*	NM_001040108.1
BLM	NM_000057.3	*MSH6*	NM_000179.2
BRD4	NM_058243.2	*MUTYH*	NM_001128425.1

BTG2	NM_006763.2	*MYOD1*	NM_002478.4
CBFB	NM_022845.2	*NCOR2*	NM_006312.5
CCNE1	NM_001238.3	*NFKBIA*	NM_020529.2
CDC73	NM_024529.4	*NOTCH3*	NM_000435.2
CDK6	NM_001145306.1	*NSD1*	NM_022455.4
CDKN2A	NM_000077.4	*NTRK1*	NM_001007792.1
CHD1	NM_001270.2	*P2RY8*	NM_178129.4
CIC	NM_015125.4	*PARP1*	NM_001618.3
CSF3R	NM_156039.3	*PCDH11X*	NM_032968.4
CTNNA1	NM_001323982.1	*PDK1*	NM_001278549.1
CYLD	NM_015247.2	*PIK3C3*	NM_002647.3
DDR1	NM_013994.2	*PIK3R1*	NM_181523.2
DNMT1	NM_001130823.2	*PLK2*	NM_006622.3
EED	NM_001308007.1	*POLE*	NM_006231.3
EP300	NM_001429.3	*PPP6C*	NM_001123355.1
EPHA7	NM_004440.3	*PRKDC*	NM_006904.6
ERBB4	NM_005235.2	*PTPRD*	NM_002839.3
ERCC5	NM_000123.3	*RAB35*	NM_006861.6
ETV5	NM_004454.2	*RAD51B*	NM_133509.3
FANCA	NM_000135.2	*RAF1*	NM_002880.3
FANCG	NM_004629.1	*RECQL4*	NM_004260.3
FAT1	NM_005245.3	*RICTOR*	NM_001285439.1
FGF19	NM_005117.2	*RPS6KA4*	NM_003942.2
FGF7	NM_002009.3	*RUNX1T1*	NM_001198679.1
FH	NM_000143.3	*SDHC*	NM_003001.3
FOXA1	NM_004496.3	*SH2B3*	NM_005475.2
FUBP1	NM_003902.4	*SLX4*	NM_032444.2
GATA3	NM_001002295.1	*SMARCB1*	NM_003073.4
GLI1	NM_005269.2	*SOX10*	NM_006941.3
GPS2	NM_004489.4	*SPOP*	NM_001007226.1
H3C2	NM_003537.3	*STAT3*	NM_139276.2
HDAC2	NM_001527.3	*STK40*	NM_001282546.1
HIST1H3C	NM_003531.2	*TCF3*	NM_003200.3
HIST1H3I	NM_003533.2	*TERT*	NM_198253.2
HLA-B	NM_005514.7	*TIPARP*	NM_001184717.1
HSD3B1	NM_000862.2	*TOP1*	NM_003286.2
IDH2	NM_002168.3	*TRAF7*	NM_032271.2
IKBKE	NM_014002.3	*TSHR*	NM_000369.2
INHBA	NM_002192.3	*VEGFB*	NM_003377.4
IRF4	NM_002460.3	*XIAP*	NM_001167.3
JAK3	NM_000215.3	*YES1*	NM_005433.3
KDM6A	NM_001291415.1	*ZNRF3*	NM_001206998.1

Table S2. Differential mutated genes

Gene Symbol	*TP53* pathologic mutation	Non-*TP53* pathologic mutation	p value
GATA3	0	11	4.52E-05
CDH1	0	6	0.00519
SDHAF2	6	0	0.036732
PALB2	2	6	0.072588
CARD11	0	3	0.076054
CHD4	0	3	0.076054
MSH3	0	3	0.076054
SF3B1	0	3	0.076054
POLE	8	1	0.076129
FANCA	3	7	0.096587
KIT	3	7	0.096587
GRM3	4	0	0.134028
MLH1	4	0	0.134028
TSC2	2	5	0.136761
CBL	0	2	0.181598
CDKN1B	0	2	0.181598
CTCF	0	2	0.181598
ESR1	0	2	0.181598
RARA	0	2	0.181598
TGFBR2	0	2	0.181598
TOP2A	0	2	0.181598
FGFR2	3	0	0.25908
FLCN	3	0	0.25908
GATA2	3	0	0.25908
SDHB	3	0	0.25908
SMAD4	3	0	0.25908
CHEK2	3	5	0.285745
BRCA1	7	2	0.297234
BLM	1	3	0.312362
FGFR1	1	3	0.312362
MAP2K4	4	1	0.390391
SPTA1	4	1	0.390391
ATM	3	4	0.459984
PRKDC	6	2	0.463691
ABL2	2	0	0.506053
BRIP1	2	0	0.506053
CUL3	2	0	0.506053
DCUN1D1	2	0	0.506053
EED	2	0	0.506053
EP300	2	0	0.506053
FANCG	2	0	0.506053
HIST1H2BD	2	0	0.506053

KDM5C	2	0	0.506053
LATS2	2	0	0.506053
MET	2	0	0.506053
MYC	2	0	0.506053
NF2	2	0	0.506053
NFE2L2	2	0	0.506053
PARP1	2	0	0.506053
PCDH11X	2	0	0.506053
PIK3R2	2	0	0.506053
PTPRD	2	0	0.506053
PTPRS	2	0	0.506053
RICTOR	2	0	0.506053
RPTOR	2	0	0.506053
SDHD	2	0	0.506053
STAG2	2	0	0.506053
STAT3	2	0	0.506053
VEGFB	2	0	0.506053
XPO1	2	0	0.506053
BRAF	1	2	0.575712
KMT2A	1	2	0.575712
KMT2D	1	2	0.575712
LYN	1	2	0.575712
PMS2	1	2	0.575712
PREX2	1	2	0.575712
PIK3CA	32	21	0.578596
ARID1A	3	1	0.634235
GNAS	3	1	0.634235
NOTCH1	3	1	0.634235
AKT1	2	3	0.650138
HIST1H1C	2	3	0.650138
MSH2	2	3	0.650138
BARD1	4	2	0.699241
MAP3K1	4	2	0.699241
PTCH1	4	2	0.699241
SMARCA4	4	2	0.699241
ERBB2	6	3	0.730677
CDK12	5	5	0.742919
BRCA2	6	6	0.759997
MSH6	5	4	1
RAD54L	5	4	1
APC	6	4	1
ATRX	2	1	1
BAP1	3	2	1
CDKN2A	2	2	1
CREBBP	2	1	1

DICER1	3	3	1
EGFR	3	3	1
FANCI	3	3	1
FLT3	2	1	1
FOXA1	2	1	1
GRIN2A	2	2	1
IRS1	2	1	1
KEAP1	2	1	1
LRP1B	3	2	1
MAGI2	2	2	1
NF1	5	3	1
NOTCH3	2	1	1
NRAS	2	1	1
NTHL1	2	1	1
PTEN	5	3	1
RB1	5	3	1
RECQL4	3	2	1
RET	2	1	1
ROS1	2	2	1
RUNX1T1	2	2	1
SDHA	3	2	1
SOX9	3	2	1
TERT	2	1	1
